# Twist1 mediated regulation of glioma tumorigenicity is dependent on mode of mouse neural progenitor transformation

**DOI:** 10.18632/oncotarget.22593

**Published:** 2017-11-21

**Authors:** Andrei M. Mikheev, Svetlana A. Mikheeva, Mari Tokita, Liza J. Severs, Robert C. Rostomily

**Affiliations:** ^1^ Department of Neurological Surgery, Houston Methodist Hospital and Research Institute, Houston, Texas, USA; ^2^ Department of Neurological Surgery and Institute for Stem Cell and Regenerative Medicine, University of Washington, Seattle, Washington, USA; ^3^ Division of Medical Genetics, University of Washington, Seattle, Washington, USA; ^4^ Department of Physiology and Biophysics, University of Washington, Seattle, Washington, USA

**Keywords:** Twist1, glioma, invasion, p53, Cre recombinase

## Abstract

Twist1 is a master regulator of epithelial mesenchymal transition and carcinoma metastasis. Twist1 has also been associated with increased malignancy of human glioma. However, the impact of inhibiting Twist1 on tumorigenicity has not been characterized in glioma models in the context of different oncogenic transformation paradigms. Here we used an orthotopic mouse glioma model of transplanted transformed neural progenitor cells (NPCs) to demonstrate the effects of Twist1 loss of function on tumorigenicity. Decreased tumorigenicity was observed after shRNA mediated Twist knockdown in HPV E6/7 Ha-RasV12 transformed NPCs and Cre mediated Twist1 deletion in Twist1 fl/fl NPCs transformed by p53 knockdown and Ha-RasV12 expression. By contrast, Twist1 deletion had no effect on tumorigenicity of NPCs transformed by co-expression of Akt and Ha-RasV12. We demonstrated a dramatic off-target effect of Twist1 deletion with constitutive Cre expression, which was completely reversed when Twist1 deletion was achieved by transient administration of recombinant Cre protein. Together these findings demonstrate that the function of Twist1 in these models is highly dependent on specific oncogenic contexts of NPC transformation. Therefore, the driver mutational context in which Twist1 functions may need to be taken into account when evaluating mechanisms of action and developing therapeutic approaches to target Twist1 in human gliomas.

## INTRODUCTION

Glioblastomas are lethal within 12–15 months after diagnosis in the majority of adult patients. This dismal prognosis is attributed in large part to highly invasive growth and capacity for resident glioma stem-like cells (GSCs) to drive tumor formation and progression, the latter a consequence of profound resistance to current standard of care therapies. Therefore the identification and inhibition of mechanisms which coordinately promote glioma cell invasiveness and stem cell phenotypes could be of great biologic and clinical relevance.

We previously reported that TWIST1 (TW), a critical regulator of epithelial mesenchymal transition (EMT), metastasis and stem cell phenotypes in carcinomas, also promotes mesenchymal change and invasion in glioma cells and enhances self-renewal of human glioma stem-like cells (GSC) *in vitro* [1, 2]. Although debated, accumulating clinical and experimental evidence suggests that resident neural progenitor cells (NPCs) are likely cells of origin for glioma [3]. We and others have shown that orthotopic transplantation of transformed NPCs isolated from the mouse forebrain can generate tumors that reliably recapitulate hallmark features of human gliomas [4–6]. Therefore, adaptation of these mouse models for the study of TW function in transformed NPCs could provide unique insights into the potential therapeutic relevance of TW inhibition as well as its roles in regulating glioma tumorigenicity and malignancy.

Numerous *in vivo* mouse cancer models have shown that TW function is a critical downstream effector for malignant phenotypes generated by multiple oncogenic pathways [7–14]. Collectively, these data suggest the potential importance of TW as therapeutic target. Fewer studies have reported the impact of TW loss of function on tumorigenicity but their results provide critical preliminary support for the therapeutic potential of directly targeting TW. For instance, TW inhibition abrogates malignancy of Kras and EGFR mutant and MET amplified NSCLC cells *in vitro* and *in vivo* by overriding oncogene induced senescence [9, 15] and reduces tumor growth of NSLC cells in flank xenograft model [16]. In a mouse model skin carcinoma, Twist deletion depletes normal follicular stem cells and significantly reduces carcinoma formation and keratinocyte proliferation [17]. While these studies suggest the therapeutic potential for targeting TW, similar studies of direct TW targeting have not yet been reported in mouse glioma models.

Therefore, we employed our previously reported syngeneic mouse glioma model [4, 5] to investigate the oncogenic contexts in which TW inhibition may impact tumorigenicity. We achieved malignant transformation of adult mouse forebrain NPCs with three transformation paradigms; co-expression of HPV E6/E7 and Ha-RasV12 (HPV/Ras), shRNA mediated knockdown of p53 and expression of Ras (shp53/Ras) and co-expression of myristoylated Akt and Ras (Akt/Ras). These transformation paradigms utilize canonical deregulated signaling pathways, p53 (HPV and p53 knockdown), Rb (HPV) and RTK/RAS PI3K (Akt and Ras) identified in human GBM [18]. Our studies demonstrated a significant effect of TW loss of function to reduce tumorigenicity in the HPV/Ras and shP53/Ras models but not in the Akt/Ras transformation paradigm. The dependence on transformation paradigms for TW mediated regulation of tumorigencity may have implications for the development of TW targeted therapies in the contexts of specific oncogenic driver mutations.

## RESULTS

### Knockdown of TW in HPV/Ras transformed NPCs inhibits tumorigenicity

**Figure 1:**
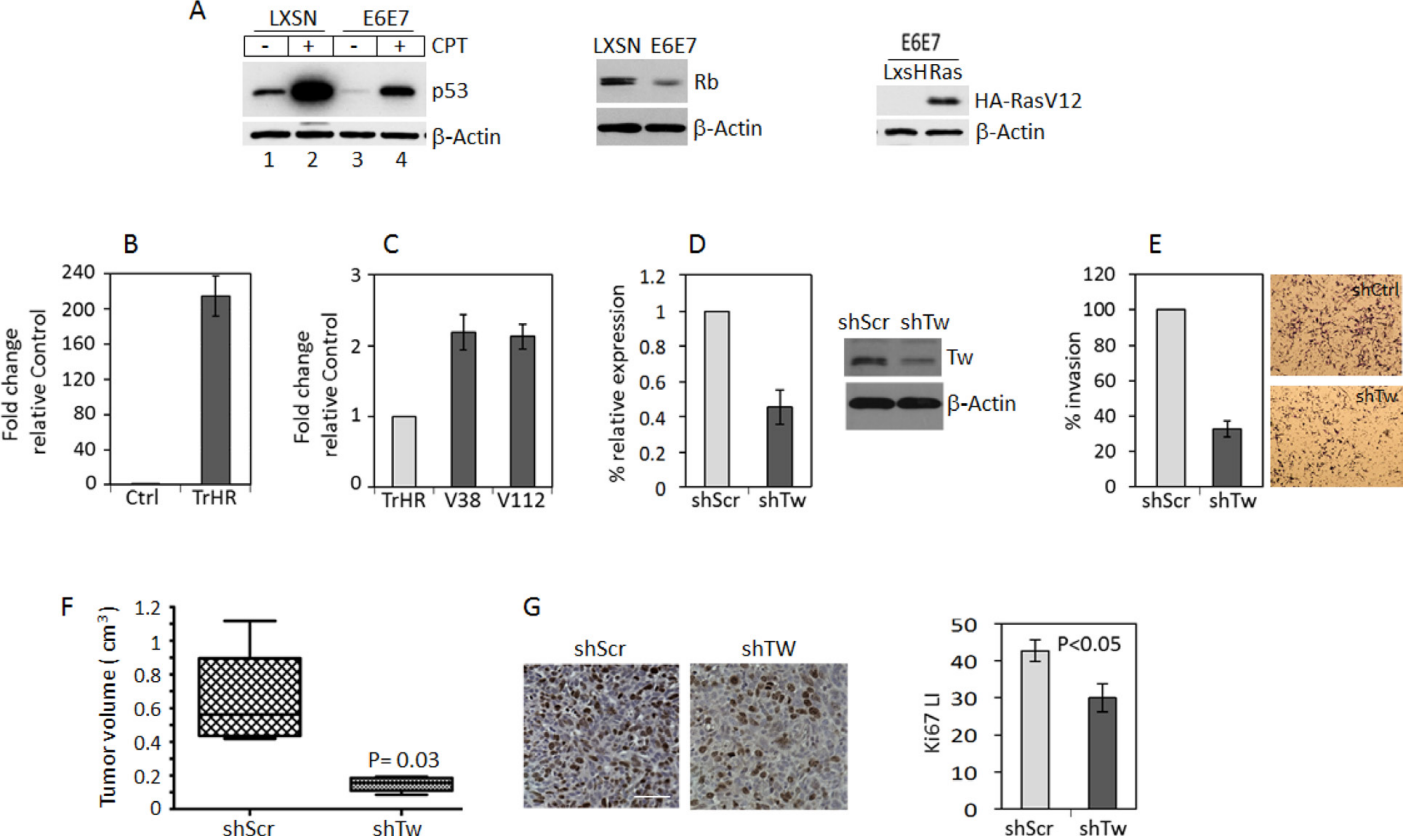
TW knockdown in HPV E6/7 transformed neural progenitor cells (NPCs) inhibits tumorigenicity (**A**) Protein expression of p53, Rb and HA-RasV12 in control and HPV E6E7 HA-RasV12 transformed NPCs. With constitutive expression of E6E7, expression and induction of p53 expression by CPT is reduced (left panel) and Rb expression is decreased (middle panel). The levels of HA-RasV12 expression are shown (right panel). (**B**) Quantification of TW mRNA after transformation of 3 month old mouse forebrain NPCs with HPV/Ras (TrHR) vs NPC transduced with empty vectors. (**C**) Cells isolated from HPV/Ras generated gliomas (V38 and V112) propagated *in vitro* show a modest 2-fold increase in TW expression compared with the parental cells (TrHR) prior to implantation. (**D**) shRNA specific for TW (shTw) produced a 60% knockdown of TW mRNA expression in V38 cells. Corresponding TW protein expression levels measured by Western Blot are shown (right). (**E**) Invasion of V38 cells after TW knockdown quantified in a matrigel invasion assay. (**F**) Equal numbers of V38 control and shTW cells were injected orthotopically and tumor volumes were quantified at 40 days post-injection. (**G**) Representative Ki-67 immunohistochemistry of glioma sections from control (left panel) and shTW (right panel) generated tumors. Scale bar, 30 μm. Quantification of Ki-67 labeling indices form randomly selected fields shows a significant decrease in proliferation of shTW tumors (*p* = 0.03).

Using previously generated and characterized HPV/Ras transformed NPCs derived from 3 month-old mouse forebrain [5] we verified alterations in basal and inducible levels of p53 expression, decreased Rb expression and Ha-RasV12 overexpression (Figure 1A). After transformation we observed a marked increase in TW mRNA expression compared to vector control NPCs (Figure 1B, for protein expression see Figure 7B). Cells grown from these tumors (V38 and V112) under serum-free stem cell conditions exhibited persistently increased TW expression approximately 2-fold greater than the parental HPV/Ras transformed cells (TrHR) before implantation (Figure 1C). In the V38 tumor derived cell line we achieved approximately 60% knockdown of TW expression using a TW-specific shRNA lentivirus. (Figure 1D). Consistent with its function in human gliomas cells, knockdown of TW expression in V38 resulted in an approximately 70% decrease in cell invasion (Figure 1E). To confirm the relationship between this TW regulated phenotype and tumorigenicity we orthotopically implanted 2 × 10^5^ V38 cells into syngeneic hosts. At 40 days after implantation the mean tumor volume for V38 shTW derived tumors (0.2 cm^3^) was significantly reduced compared with those generated from V38 shScr cells (0.6 cm^3^; *p* = 0.03) (Figure 1F). The slower growth rates of V38 shTW tumors were associated with an approximately 30% reduction in cell proliferation as measured by Ki-67 labeling indices (Figure 1G). These studies demonstrated that direct inhibition of TW expression in HPV/Ras transformed NPCs markedly reduced invasion *in vitro* and tumor growth *in vivo*, in part through decreased cell proliferation. HPV E6/E7 expression inhibits key signaling nodes in GBM (p53 and Rb/P16, respectively) but HPV *per se* is not pathogenic in human GBM. Therefore, we next employed additional transformation paradigms to test the effect of TW loss of function on tumorigenicity.

### TW floxed transgenic model to determine the effects of TW knockout on tumorigenicity

**Figure 2:**
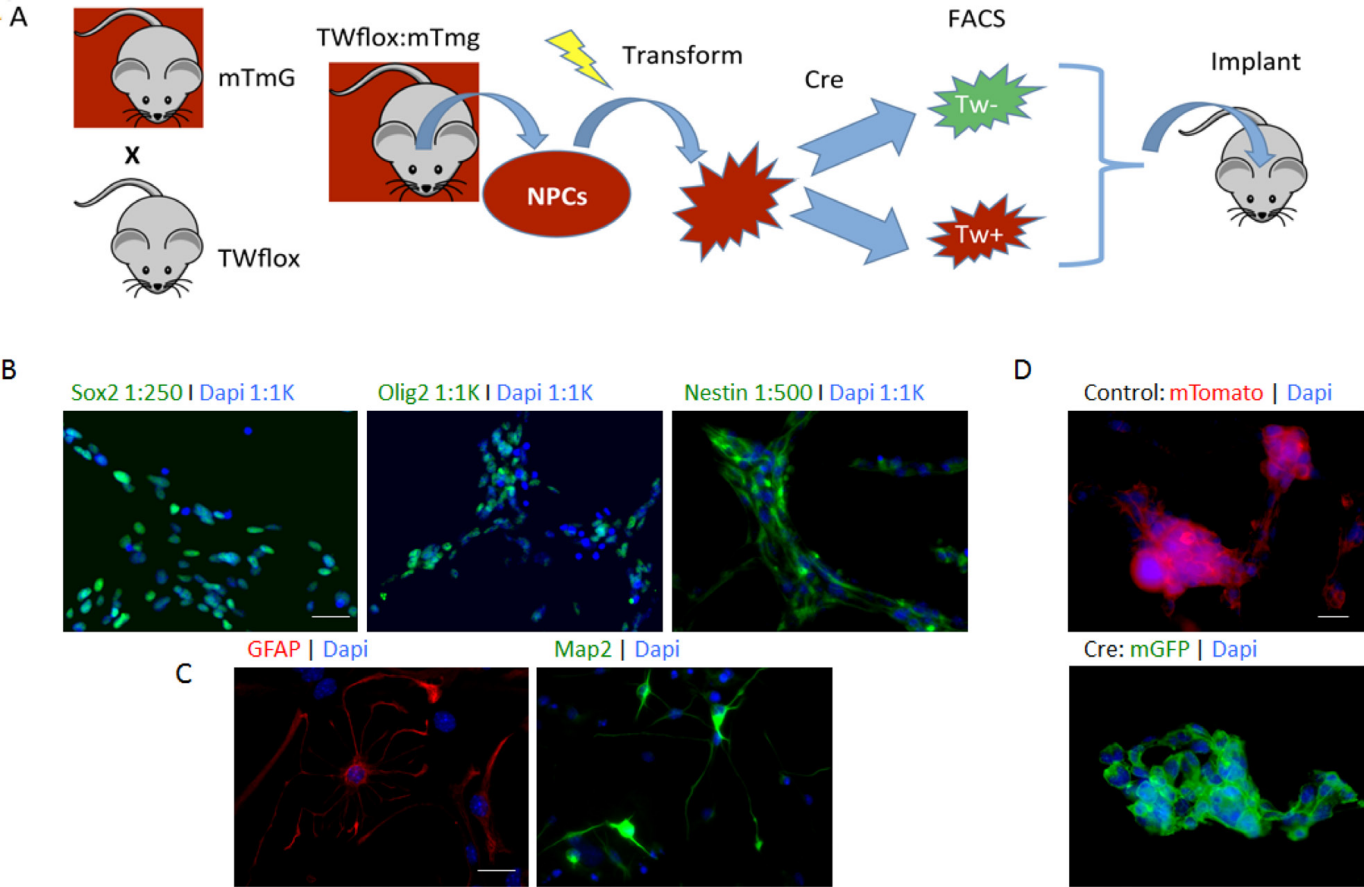
Floxed TW mouse glioma model (**A**) Scheme used to investigate TW function in TWflox:mTmG transgenic mice. (**B**) Immunophentyping of NPCs prior to transformation showing expression of neural stem and progenitor markers Sox2, Olig2 and nestin. Scale bar, 30 μm. (**C**) NPCs are multi-potent expressing astrocytic (GFAP) and neuronal (Map2) markers after exposure to differentiating conditions. Scale bar, 20 μm. (**D**) Conversion of mTmG Cre reporter after exposure to Cre recombinase. Scale bar, 10 μm.

In the above experiments using shRNA we achieved partial knockdown of TW. In order to ascertain the absolute functional requirements of TW in transformed NPCs we employed a TW floxed transgenic model in which we could achieve complete knockout of TW expression. To provide a surrogate reporter of TW recombination and knockout we crossed TW floxed transgenic mice with mTmG mice (TWflox:mTmG). After exposure to cre recombinase and successful recombination at loxP sites, cells harboring the mTmG locus convert from Tomato red membrane fluorescence to GFP membrane fluorescence. This provides a readout for cre-mediated recombination at the TW loxP loci as well. Neural progenitor cells (NPCs) were isolated from the forebrain of 3 month old Twflox:mTmG transgenic mice (Figure 2A) as previously reported for C57Bl/6 mice [4]. The TWflox:mTmG forebrain NPCs expressed neural progenitor markers Sox2, Olig2 and Nestin when cultured in proliferation media and markers of astrocytes (GFAP) and neurons (Map2) under differentiating conditions (Figure 2B, 2C). Stable expression of cre recombinase also resulted in a switch from membrane Tomato to green fluorescent protein expression (Figure 2D). Therefore, these cells were confirmed to possess immunophenotypes and differentiation potential consistent with NPCs and conversion from red to green membrane fluorescence upon exposure to cre recombinase.

### Knockout of TW by constitutive expression of Cre recombinase dramatically inhibits tumorigenesis of Akt/RAS transformed NPCs

**Figure 3:**
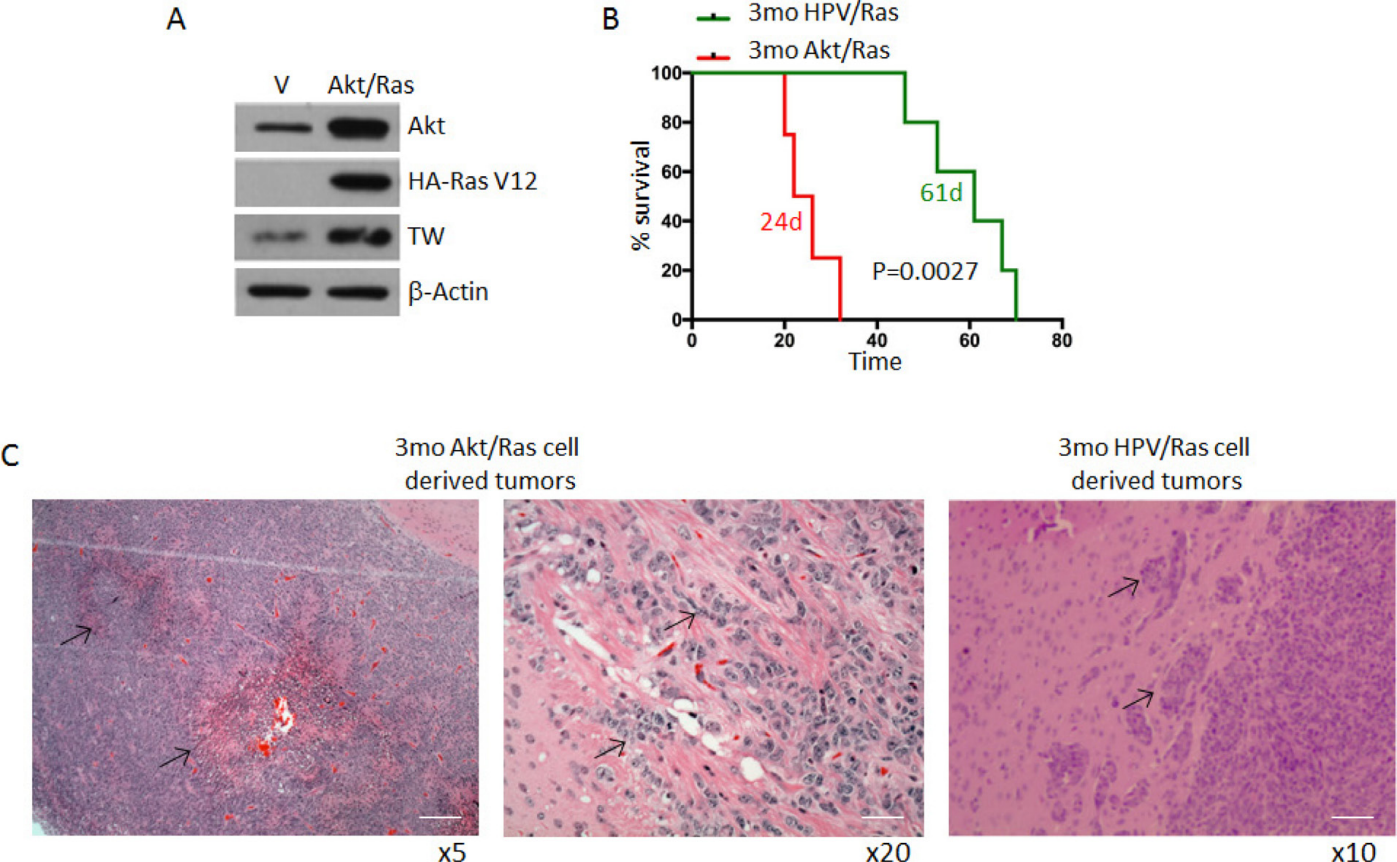
Comparative survival of 3mo mouse NPCs transformed by HPV/Ras versus Akt/Ras (**A**) Transformation of NPCs with Akt/Ras resulted in upregulation of TW compared with NPCs transfected with empty vectors as control. (**B**) Median survival of animals implanted with equal numbers of HPV/Ras (61 days) and Akt/Ras (24 days) (*p* = 0.0014). (**C**) Representative histology from Akt/Ras cells showing marked pseudo-palisading necrosis (left image, arrows). Higher magnification demonstrates invasive cells in white matter. Scale bar, 100 μm (left), 25 μm. HPV/Ras tumors demonstrated local invasion only (arrows) without necrosis Scale bar, 50 μm.

According to the TCGA analysis, deregulation of the RTK/RAS/PI3K/Akt signaling node occurs in 88% of adult GBMs [18]. Mouse glioma models based on co-activation of Ras and Akt have been shown to produce gliomas that closely phenocopy human GBMs [6]. To compare the tumorigenicity of this paradigm with HPV/Ras transformed NPCs as shown above we co-expressed myristoylated Akt and Ras (AR) to transform adult forebrain derived NPCs from C57Bl/6 mice. TW protein expression was increased in AR cells compared to control NPCs (Figure 3A) and survival of animals implanted with AR transformed NPCs was markedly reduced compared to animals implanted with age matched HPV/Ras transformed NPCs (Figure 3B; median survival of 24 versus 61 days; *p* = 0.0027). Furthermore AR cell-derived tumors exhibited hallmark pathologic features of human GBM including pseudopalisading necrosis, which was not observed in tumors derived from HPV/Ras transformed cells (Figure 3C). Therefore, the AR model recapitulated key features of human GBM.

**Figure 4:**
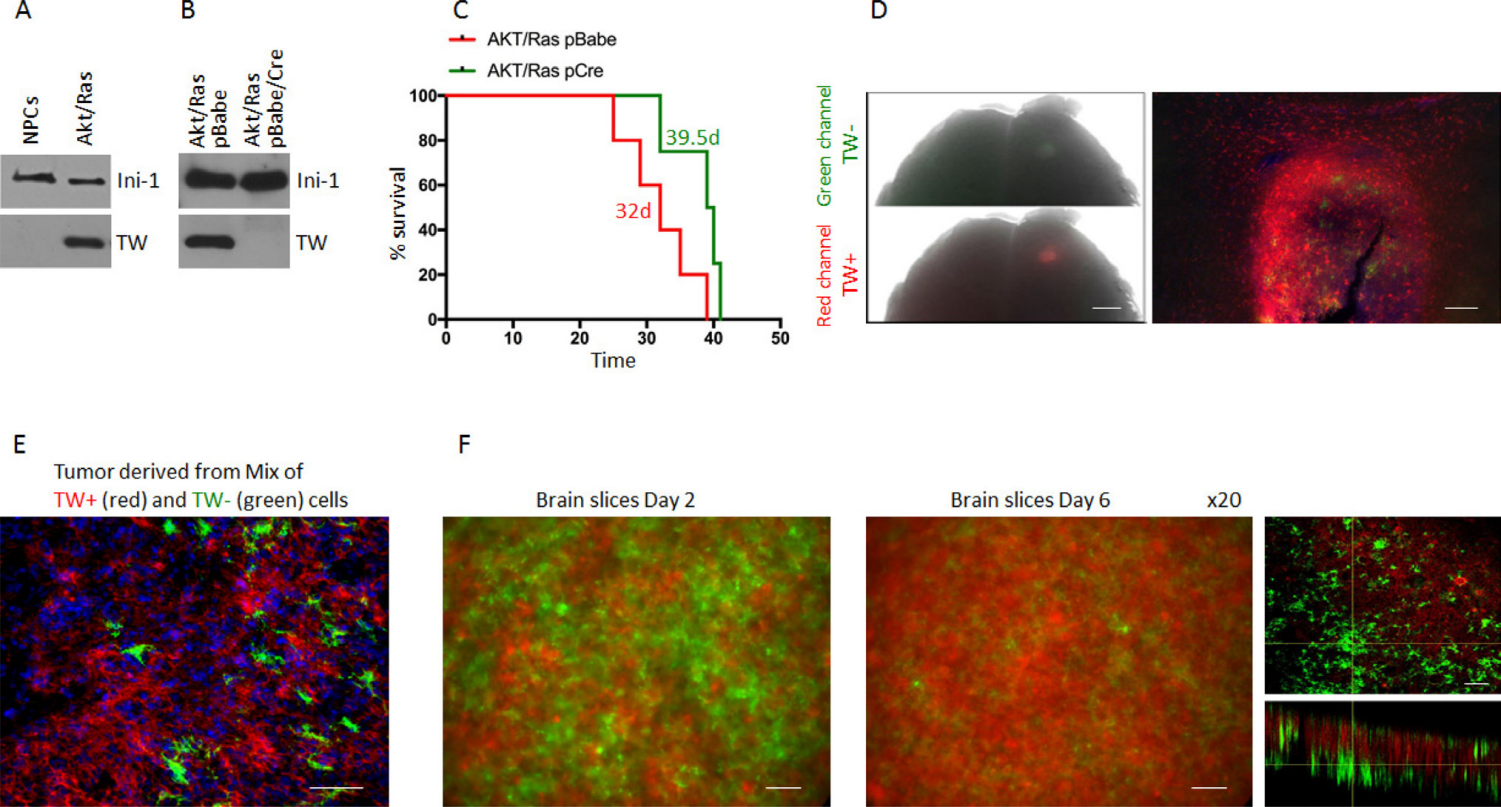
Outgrowth of TW+ cells after implantation of unsorted Akt/Ras NPCs infected with Cre recopmbinase (**A**) Western blot of NPCs co-expressing Akt/Ras upregulate TW expression. (**B**) After stable infection with Cre recombinase green conversion was >98% (not shown) and no TW protein was detected by western blot. (**C**) Survival of animals implanted with the Cre infected cells was prolonged (Survival time is shown for each group, *p* = 0.064). (**D**) Example of whole brain image of animal brain 10 d after implantation of unsorted primarily green Cre cells. Both green and red channel fluorescence are detected under dissecting microscope. Scale bar, 1 mm. Histologic image (10×) of the same tumor comprised nearly entirely of red TW+ cells (right panel) capable of invading brain tissue. TW null cells are detected in the tumor mass, but no invasion of GFP positive cells is detected. Scale bar, 50 μm. (**E**) Photomicrograph of tissue section from tumor after 50:50 implantation of TW+ (red) and TW− (green) cells shows nearly complete loss of TW− cells. Scale bar, 20 μm. (**F**) Temporal changes in cell survival after seeding equal numbers of TW+ (red) and TW− (green) on a postnatal mouse brain section. Serial images of a representative experiment after 2 days in culture (left) and 6 days in culture (right). Representative confocal image of the brain slice after 6 days of culture clearly demonstrate outnumbering of TW+ (red) cells. Scale bar, 30 μm.

**Figure 5:**
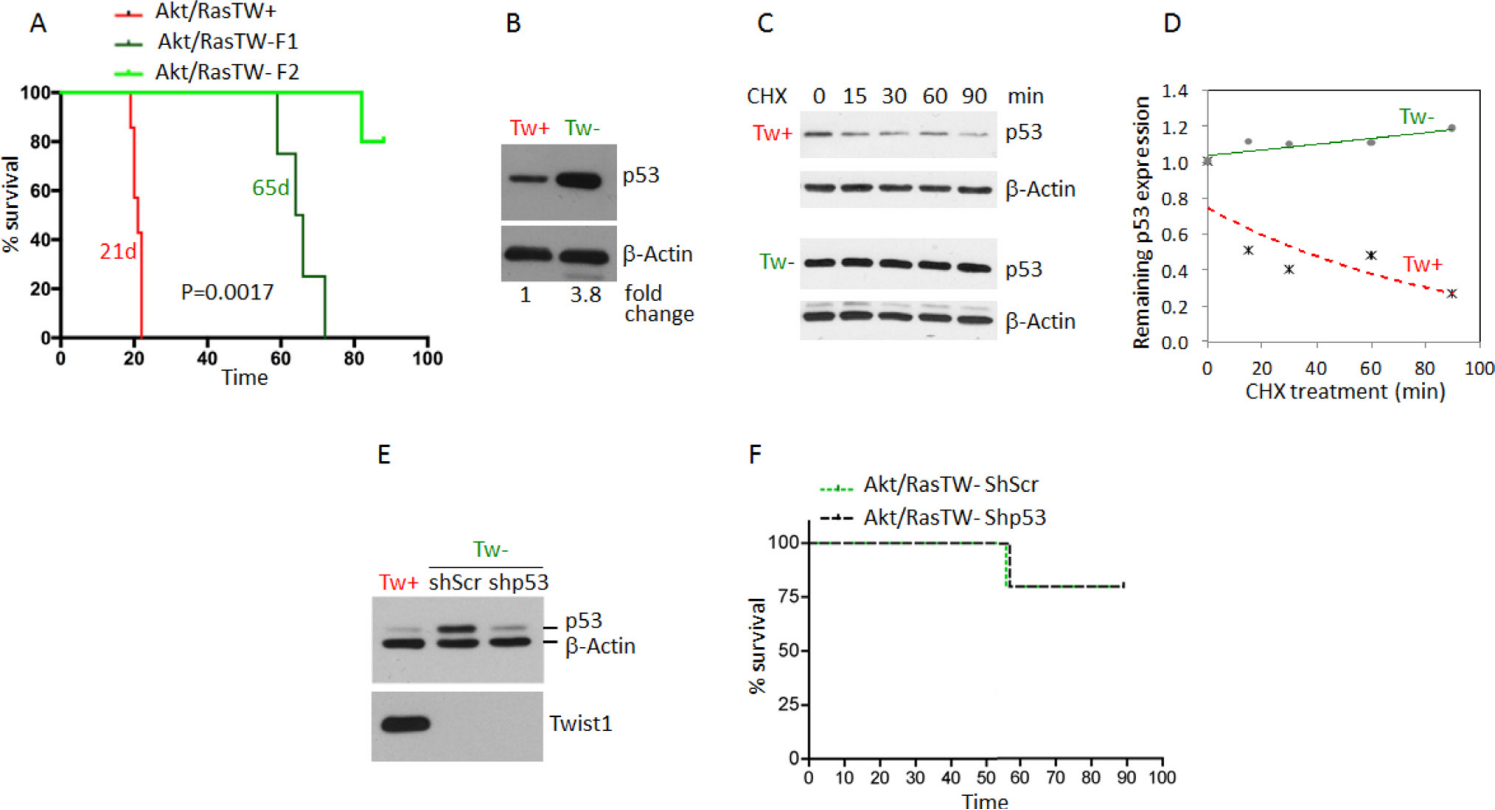
Deletion of TW in Akt/Ras transformed TWflox:mTmG NPCs by constitutive Cre expression nearly completely inhibits tumorigenesis independent of p53 stabilization (**A**) Marked increase in the survival of nude hosts after implantation of Tw null (green) versus Tw+ control cells (red). To eliminate contaminating Tw+ cells serial FACS was performed (F1 then F2). The more stringent F2 (no detectable red cells) resulted in the greatest increase in animal survival. (**B**) TW deleted Akt/Ras NPCs (TW–) demonstrated significant upregulation of p53 compared with TW+ control cells. (**C**) Stability of p53 protein in TW+ and TW– cells. (**D**) Degradation curves show dramatic stabilization of p53 in Cre TW– cells. (**E**) Lentiviral mediated knockdown of p53 in TW– cells confirmed by Western blot. (**F**) Knockdown of p53 in TW– cells does not impact survival. In each group single TW– (green) tumor was detected out of 5 animals injected within 90 days of observation.

To determine the absolute functional requirements of TW in the context of Akt/Ras we transformed NPCs from the forebrain of 3 month old Twflox:mTmG transgenic mice by co-expression of myristoylated Akt and oncogenic Ha-RasV12 (Akt/Ras). Upon transformation we observed a marked upregulation of TW as shown above (Figure 4A). To delete TW we split the Akt/Ras transformed NPCs into two cultures and infected one with a pBabe Cre recombinase retroviral expression vector and the other with pBabe empty vector as a control. After selection, GFP expressing TW deleted cells comprised >98% of the cell culture (not shown) and TW expression was not detectable by western blot analysis (Figure 4B). We then implanted equal numbers of the AR pBabe and AR pBabe/Cre cells into syngeneic littermates and observed a modest improvement in survival in animals implanted with the TW deficient AR transformed NPCs (Figure 4C). However, gross examination of these tumors at sacrifice revealed that they were entirely composed of dTomato expressing cells indicating TW expressing cells (TW+) (not shown). Analysis of tumors 10 days after implantation demonstrated residual TW deficient GFP+ cells although the vast majority of the tumor cells expressed dTomato indicating *in vivo* selection for rare TW+ cells from the original implanted cell population (Figure 4D). The presumed selection advantage of TW+/dTomato+ cells was confirmed using equal mixtures of TW+/dTomato+ and TW–/GFP+ cells grown as orthotopic implants (Figure 4E) and co-cultures on organotypic brain slices (Figure 4F). We concluded that the minor population of TW+/dTomato+ cells outgrew the TW deficient cells. To confirm this selection effect we performed serial FACS (F-1 and F-2) gated to increasingly eliminate contamination from TW+ cells (not shown). We observed a dramatic decrease in tumorigenicity from TW– cell populations proportional to the increasing stringency of F-1 and F-2 gating for residual TW+ cells (Figure 5A). To determine whether the apparent role of TW to promote tumorigenicity in AR transformed NPCs is associated with its capacity to accelerate degradation of p53 [19] we studied p53 expression and stability. We found that p53 expression and stability were dramatically increased in TW– cells compared with Tw+ cells (Figure 5B–5D). To test the dependence of the growth inhibition of TW– cells on p53 we knocked down p53 in the TW– Akt/Ras NPCs to the levels observed in TW+ cells (Figure 5E). Unexpectedly, p53 knockdown had no effect on the tumorigenicity of the TW– cells (Figure 5F). This result suggested that the effects of TW on tumorigencity of Akt/Ras transformed cells is not dependent on p53. However, another consideration is whether the use of constitutive cre expression to achieve recombination resulted in an off-target effect.

### Deletion of TW in Akt/Ras transformed NPCs with recombinant Cre protein does not inhibit tumorigenicity

**Figure 6:**
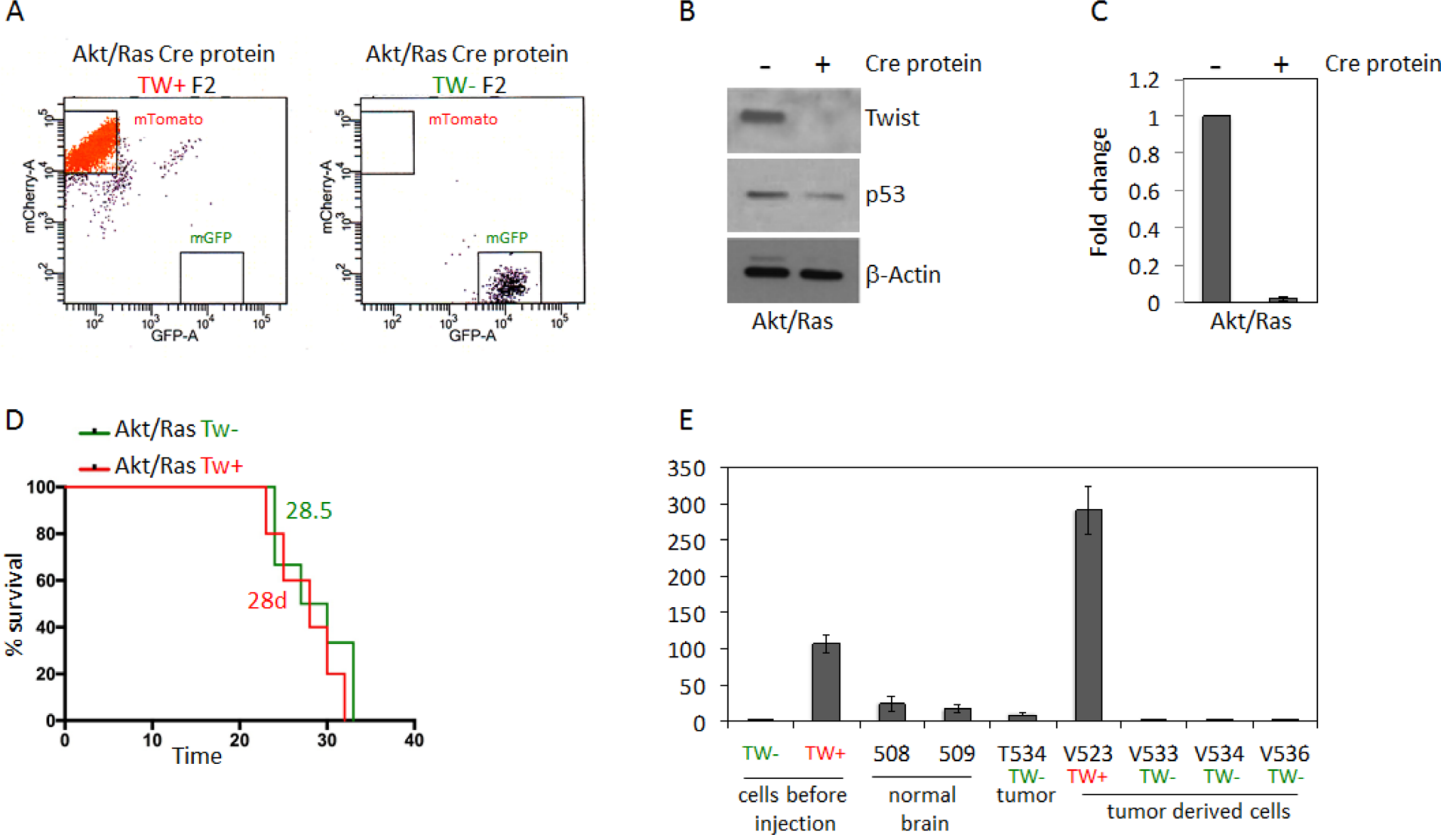
Deletion of TW by recombinant Cre recombinase transfection in Akt/Ras transformed NPCs does not impact survival (**A**) FACS analysis of cells after second FACS (F2) enrichment shows pure populations of mTomato (TW+) and GFP (TW–) cells. (**B**) Western blot analysis of F2 whole cell lysates shows no detectable TW protein and reduction of steady state p53 expression after Cre treatment. (**C**) Lack of TW mRNA confirmed by RT-PCR. (**D**) After implantation of equal numbers of Akt/Ras transformed TW WT and TW null NPCs no difference in animal survival is noted. (**E**) RT-PCR analysis of TW expression in TW– tumor derived cells (V533, V534, V536) demonstrates retention of TW deletion in TW negative cells compared to TW positive control V523. Example of low TW expression in TW negative tumor (T534) remains below TW expression in normal brain (508, 509). Tw expressions in cells before injection are shown.

To address potential off-target effects of constitutive cre recombinase expression we analyzed the efficacy of transient exposure to recombinant cre protein in cell cultures to generate recombination. With 5 uM recombinant cre protein in culture we achieved 72% conversion rate from dTomato to GFP after 6 days in culture (Supplementary Figure 1). With serial FACS (F2) we isolated pure populations of TW + (red) or TW– (green) AR NPCs (Figure 6A) and confirmed the absence of TW protein and mRNA in the GFP selected cells after exposure to cre (Figure 6B, 6C). Of note, basal p53 expression was decreased in these cells (Figure 6B) in contrast to the increased basal expression and stabilization observed in the same cells harboring constitutive Cre (Figure 5B–5D). Remarkably, when using transient Cre recombinase treatment, TW knockout had no effect on tumorigenicity (Figure 6D). TW knockout was confirmed in tumor and tumor derived cells (Figure 6E). These studies strongly suggested that the anti-tumor effects of TW deletion observed when using constitutive Cre recombinase were most likely due to persistent Cre expression rather than a specific effect of TW deletion on tumorigenicity.

### Tumorigenicity of NPCs transformed by p53 knockdown and Ras is TW dependent

**Figure 7:**
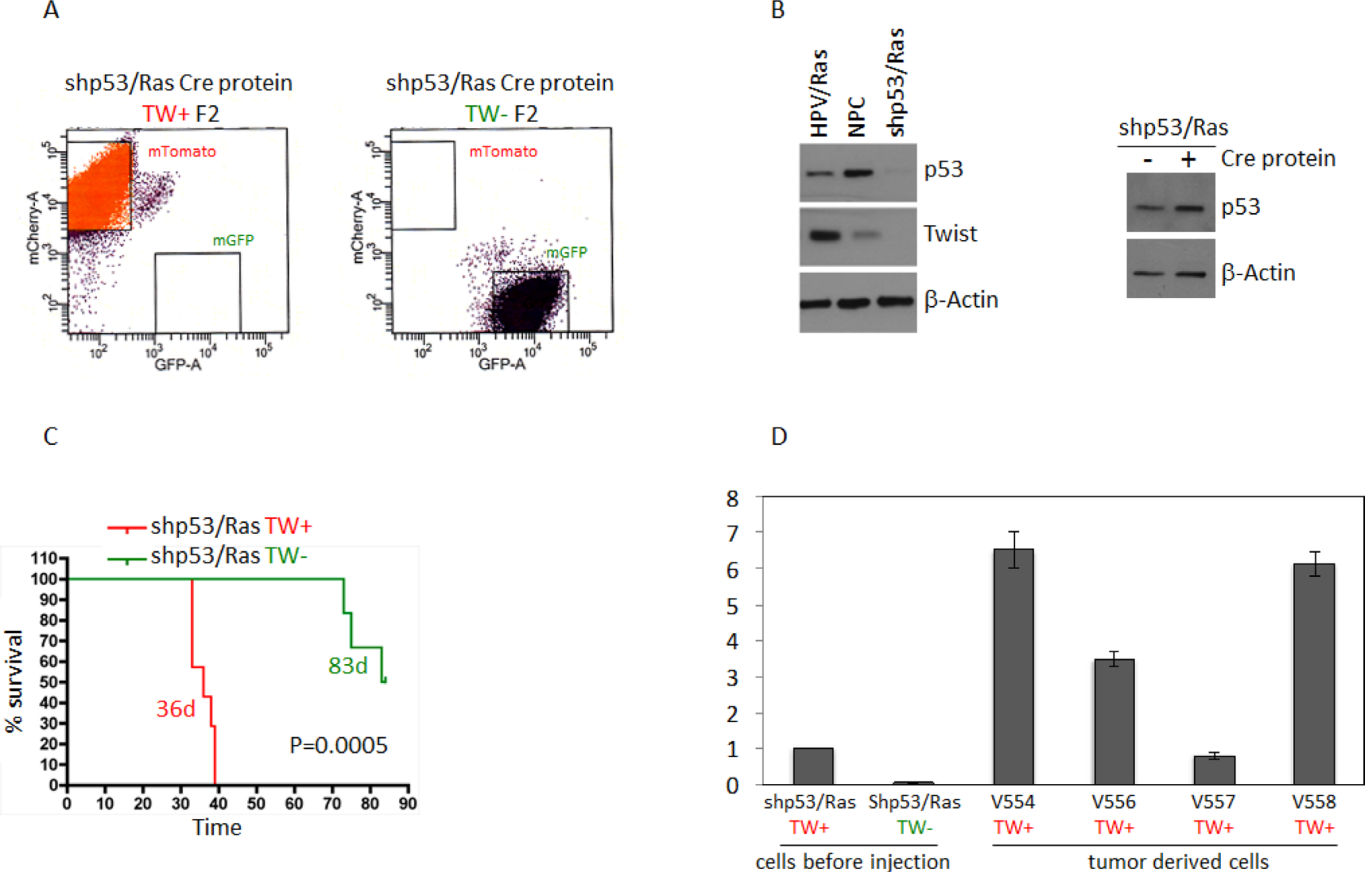
Deletion of TW in shP53/Ras transformed NPCs prolongs survival (**A**) FACS enrichment (F2) shows pure populations of mTomato (TW+) and GFP (TW−) cells. (**B**) Levels of p53 expression in cells transformed with shp53/Ras compared to normal NPCs and cells transformed with HPV/Ras. Ha-RasV12 expression not shown. Levels of TW in shp53/Ras cells is below detection limit on the Western blot compared to normal NPCs or HPV/Ras transformed cells. After Cre mediated TW deletion levels of p53 expression are not changed. TW deletion is confirmed by fluorescence conversion and qRT-PCR (shown in panel D) (**C**) Deletion of TW prolongs survival of hosts implanted with shP53/Ras transformed NPCs. Tumors were verified for fluorescence corresponding to TW genotype. No contamination was found in TW null (green) tumors. Animals sacrificed without clinical manifestation demonstrated surviving TW– cells/small tumor at injection site (Supplementary Figure 2). (**D**) TW mRNA expression in shP53/Ras Cre cells derived from tumor showing increased TW expression after growth *in vivo*.

In addition to the RTK/RAS/PI3K signaling axis, p53 is a hallmark pathway deregulated in 87% of GBMs [18]. As shown above, TW knockdown resulted in a reduction in tumorigenicity with HPVE6/7 where both p53 and Rb signaling are inhibited. Here, we sought to interrogate the role of TW knockout in the setting of p53 loss of function alone in NPCs transformed through shRNA lentiviral mediated knockdown of p53 and over-expression of Ras (shP53/Ras). We deleted TW in TWfloxed:mTmG shP53/Ras transformed NPCs by administering recombinant cre recombinase in culture (Supplementary Figure 1A, 1B) and performing two rounds of sequential FACS isolation (F2) to enrich for pure populations of TW+ (red) and TW– (green) cells (Figure 7A). A marked decrease in p53 expression was achieved compared with HPV/Ras transformed and normal NPCs (Figure 7B). Surprisingly, unlike in HPV/Ras or Akt/Ras transformed NPCs, TW expression was not detectable *in vitro* by western blot after transformation with shP53/Ras compared normal cells (Figure 7B, left). Steady state levels of p53 expression in shP53/Ras transformed cells could be detected by prolonged exposure of western blots and no apparent changes were noted in basal p53 expression due to administration of recombinant Cre protein (Figure 7B, right). This indicated that recombinant cre protein used to delete TW in shP53/Ras transformed cells did not increase p53 expression as seen with constitutive cre in AR transformed cells above. Despite the lack of detectable TW expression acutely after transformation animals implanted with shP53/Ras transformed NPCs selected for mTmG conversion and TW gene knockout exhibited a dramatic prolongation of median survival compared to those implanted with control shP53/Ras transformed NPCs without TW gene knockout (83 versus 36 days; *p* = 0.0005; Figure 7C). Examination of the resulting tumors for each genotype demonstrated expression of mTomato or mGFP demonstrating the retention of the original TW genotype (Supplementary Figure 2). To reconcile the robust impact of TW deletion with its lack of upregulation acutely after transformation we hypothesized that TW upregulation may occur *in vivo* and confer a growth advantage to TW competent cells. To test this hypothesis, we compared levels of TW mRNA expression in tumor derived cells isolated from 4 different shP53/Ras tumors (TW+) with the pre-implanted shP53/Ras transformed NPCs. In 3 of 4 tumor derived cell isolates TW expression was increased at approximately 3, 6, and 6.5-fold compared with parental cells with one cell line exhibiting no change (Figure 7D). These data suggest that TW may contribute to the tumorigenicity of shp53/Ras transformed NPCs through temporal up-regulation during tumor progression *in vivo*. The degree of TW upregulation seen in the tumor derived cells, and absence of an increase in one line, suggest that even modest increased in TW expression may be critical to support tumor growth and progression *in vivo*.

## DISCUSSION

We previously showed that TW promotes malignant properties of human glioma cells *in vitro* [1, 2]. However, the potential importance of TW as a therapeutic target and the oncogenic contexts in which this may be relevant have not been studied in glioma. Here we addressed this question using adult mouse forebrain derived NPCs transformed with three separate paradigms in a syngeneic orthotopic transplant model. We showed that TW loss of function led to transformation paradigm specific effects on host animal survival. Of note, paradigms that included abrogation of p53 function as part of the transformation strategy demonstrated a survival benefit for TW loss of function while TW deletion in NPCs transformed by Akt and Ras had no observable benefit for survival. To our knowledge this is the first example of a transformation paradigm dependent effect of TW inhibition on tumorigenicity in a cancer model. These findings suggest diverse functional effects of TW depending on oncogenic mutational context which could be of clinical importance when devising therapeutic strategies targeting TW.

Several mechanisms have been identified by which TW may promote glioma malignancy. TW was first described as a putative oncogene on the basis of its capacity to override oncogene and p53 induced senescence in fibroblasts, the latter ascribed to down regulation of Arf [20]. Subsequently TW was shown to down regulate Arf in immortalized epithelial cells resulting in increased cell proliferation and genomic instability in part by bypassing the p53 DNA damage response [21]. Of note, a direct interaction of TW with p53 promotes p53 degradation [19, 22]. Consistent with this mechanism, repression of TW mediated inhibition of p53 through interaction with HoxA5 represses malignant breast carcinoma cell phenotypes [23]. However, studies with NSLC cell lines showed that TW was capable of overriding oncogene induced senescence independent of the p53 pathway [15]. Here, TW loss of function in Akt/Ras transformed NPCS which retain wild-type p53 had no impact on tumorigenicity suggesting that TW mediated inhibition of p53 was not functionally important in this model. Further, the inhibition of tumorigenicity in NPCs transformed in part through abrogation of p53 supports the notion that TW has functional roles independent of its inhibition of p53. With regard to the Rb pathway, TW has been implicated in its deregulation, particularly through its effect on p16 suppression, as another mechanism to override senescence [24]. These studies highlight the potential complexity of TW functions related to p53 and the need for further investigation of TW mechanisms of action in glioma cells in different oncogenic contexts.

Reciprocal regulatory interactions also exist between TW and the Akt pathways. In breast carcinoma, TW activates Akt signaling to promote maintenance of cancer stem cell phenotypes [25], metabolic reprogramming via coordinated activation of Akt signaling and repression of p53 and invasion [26] and drug resistance by upregulation of Akt2 [27]. Conversely Akt also regulates TW function through site-specific phosphorylation that can result in either stabilization or degradation [28, 29]. Of note, Akt1 has specifically been implicated in the phosphorylation dependent degradation of TW [29]. Here we used a constitutively expressed Akt1 isoform that conceivably could have rendered wild-type TW functionally inactive through degradation and account for the lack of a difference in tumorigenicity between TW+ and TW– cells. Arguing against this is that similar basal levels of TW protein are detected in wild-type Akt/Ras and HPV/Ras transformed cells. Future experimental determination of differences in TW protein degradation between the two cell types and rescue by re-expression of a TW mutant resistant to phosphorylation by Akt1 or transformation with Akt2 which is reported to stabilize TW could help resolve this question [29]. Alternatively, TW can activate AKT phosphorylation [11]. Therefore transformation by Akt over-expression may bypass the functional importance of TW as a critical upstream regulator of Akt.

Unlike a prior study in NSCLC where inhibition of TW decreased tumorigenicity in cells with different oncogenic drivers [15], we showed here that inhibition of TW had different effects on tumorigenicity depending on the mode of transformation. These disparate results may reflect the differences in models used, or potentially, species or tumor-type specific differences in TW function. Additional studies with TW inhibition in human GBM cells with different oncogenic driver mutations are needed to address this disparity.

There are several potential explanations for the transformation dependent differences in TW function observed in our models. First, the use of knock down in one model and knockout in the other two to achieve TW loss of function may result in fundamental differences in TW function; residual TW expression in the former could prevent the emergence of compensatory bypass mechanisms likely to be more relevant with complete gene deletion. However, the major difference in tumorigenicity was observed between the two TW knockout models suggesting that this alone does not account for our observations. In addition, as outlined above, the regulation and interactions between Twist and the pathways corresponding to our modes of transformation are complex and therefore unique cross-talk between these and TW are likely to contribute to our results. Aside from interactions with the oncogenic drivers, TW is likely to activate additional mechanisms unique to these different transformation contexts, which further influence tumorigenicity. This question could be addressed in future studies through comparative transcriptomic analysis.

Another interesting observation is the discordance between the level of TW up-regulation after transformation and different effects of TW loss of function on survival. For example, TW levels were markedly increased in HPV/Ras and Akt/Ras cells compared with untransformed NPCs while up-regulation of TW was detected in shP53/Ras NPCs only after their growth *in vivo*. Two factors could potentially account for this observation. First, considering the differences between the Akt/Ras and shP53/Ras models, if the marked up-regulation of TW in the former is critical for cell proliferation during the process of cell selection and growth *in vitro*, then compensatory mechanisms could be selected for to offset the loss of TW prior to implantation *in vivo*. By contrast, with shP53/Ras if activation of TW expression is critical for cell survival and malignant progression *in vivo* then the lack of TW could contribute to delayed or inefficient tumorigenesis. Second, functional redundancy of transcription factors could be operative as a compensatory mechanism unique to the Akt/Ras paradigm; a possibility that could confirmed through transcriptomic analysis of Akt/Ras cells at different stages of derivation [30–32]. Finally, the HPV/Ras and shp53 Ras paradigms share molecular mechanisms of p53 abrogation and Ras activation. Therefore, the similar effects of TW loss of function on enhanced survival in these settings could reflect a TW mediated override of Ras mediated senescence which as shown for NSLC is independent of p53 status [15].

Another important observation in this study is the dramatically different effects on tumorigenicity associated with the use of constitutive expression of Cre recombinase versus transient exposure to recombinant Cre protein. While both methods were effective in promoting deletion of TW, the phenotype generated by constitutive Cre was consistent with a Cre induced artifact rather than a specific effect of TW deletion. This conclusion was supported by the complete absence of a TW dependent effect on survival when recombinant Cre was used to induce recombination and deletion of TW. However, the possibility that deletion of TW cooperates with constitutive expression of Cre to inhibit tumorigenicity cannot be ruled out. The non-specific effect of constitutive Cre has previously been reported in a mouse lymphoma model where activation of Cre recombinase alone induced dramatic regression of p53 deficient tumors [33]. The toxic effect of Cre recombinase has been shown to correspond to dose dependent DNA damage resulting in G2/M arrest and aneuploidy [34] and may be mediated through endogenous mammalian pseudo loxP sites which can support recombination events as efficiently as bacterial derived artificial LoxP sites [35, 36]. This toxicity is dose dependent and we hypothesize that the low levels of Cre and transient exposure afforded by the use of recombinant protein can achieve recombination with minimal toxicity. It is plausible that the dose dependent effects of Cre to induce off-target DNA damage may explain the dramatic differential p53 expression and stabilization profiles observed here. The failure to rescue the phenotype with p53 knockdown may indicate that the anti-tumorigenic phenotype generated by Cre is p53 independent as suggested by the lymphoma p53 deficient model or that our knockdown was insufficient to overcome persistent Cre driving ongoing DNA damage and cell death or senescence. Our experiments with Cre mediated genetic manipulation reinforce the importance of necessary controls to exclude off-target Cre effects.

The goal of the present mouse glioma study was to demonstrate the impact of TW loss of function in different paradigms of malignant transformation of NPCs. The major findings were that TW loss of function inhibits tumorigenicity in a transformation paradigm dependent fashion and that initial levels of TW up-regulation in transformed NPCs do not correlate with the ultimate impact of TW loss of function on animal survival. These observations provide preliminary support for the therapeutic relevance of TW inhibition in human gliomas and suggest novel mechanisms of action for further study. Future studies are warranted to establish the diversity of mechanisms by which TW may promote malignancy and how they can leveraged for therapeutic benefit. For instance, validation of a compensatory mechanism in the Akt/Ras model could support the use of synthetic lethal screens against TW to short circuit a potential bypass mechanism. Finally, additional studies in human glioma cells with a range of defined molecular drivers are critical to ascertain the full clinical relevance of targeting TW.

## MATERIALS AND METHODS

### Transgenic mice and neural progenitor cell (NPC) isolation

All animal procedures were performed according to protocols approved by the University of Washington Institutional Animal Care and Use Committee. For the HPV/Ras experiments NPCs were isolated from 3mo C57/Bl6 mice as previously described [5]. For the Akt/Ras and shp53/Ras studies 129×1/SvJ mice harboring the mTmG transgene (Cre reporter) were crossed with C57Bl/6 mice homozygous for floxed TW and genotyped as described [37]. Forebrain NPCs in all cases were isolated as previously described (Petit 2007) from 3 mo old C57Bl/6 females and offspring heterozygous for mTmG and homozygous for floxed TW. The isolated cells were grown in EGF and bFGF-supplemented serum-free proliferation media (DMEM/F12 with 2 mM glutamine, 1% N2, and 50 μg/ml heparin).

### Histology and immunohistochemistry

Brains were harvested after perfusion with 4% paraformaldehyde (PFA). Paraffin embedding, sectioning and hematoxylin and eosin staining were performed at the Histology Core of the University of Washington. Unstained slides were de-paraffinized and after blocking were incubated with Ki67 rabbit antibody and goat anti-rabbit secondary antibody conjugated with HRP. Antibody binding was visualized with diaminobenzidine (DAB) (Vectorlab) and counterstained with hematoxylin. DAB positive cells and total number of cells were counted in random fields of view. Labeling index is presented as mean percent of positive cells counted in 5 independent tumors in each group of animals.

### Immunocytochemistry

For NPC characterization, 0.05 × 10^6^ cells were plated on laminin-coated glass coverslips and placed in a 24-well dish in proliferation media. After 24 hours, cells were fixed with 4% paraformaldehyde at 37°C for 10 minutes, rinsed three times with PBS, and blocked for 1 hour in PBS with 0.1% Triton X-100 and 50% Problock. Fixed cells were then subject to overnight incubation at 4°C with primary antibodies 10% problock, 0.1% Triton-X in PBS including anti-Nestin mouse monoclonal (Chemicon MAB353, 1:500, Millipore), anti-SOX2 goat polyclonal (SC17320, 1:250, Santa Cruz), and anti-Olig2 rabbit polyclonal antibody (gift from H. Yokoo [38, 39]. Secondary antibody incubation for 2 hours at room temperature was performed with antibodies diluted in PBS with Alexa 488-conjugated donkey anti-mouse, donkey anti-rabbit, and Cy2-conjugated donkey anti-goat. To characterize multipotency, cells were plated on laminin-coated coverslips in a 24-well dish. After twenty-four hours, proliferation media was exchanged for lineage specific differentiation media (5% FBS in proliferation media for astrocytic differentiation; 2% B27 and 20 ng/ml BDNF in proliferation media for neuronal differentiation). Five days later, cells were fixed and washed as above, then labeled with anti-GFAP rabbit polyclonal antibody (Z0334, 1:500, Dako) and anti-MAP2 mouse monoclonal antibody (Sigma M1406, 1:500).

### Retroviral and lentiviral virus production

For production of retroviruses and lentiviruses ecotropic Phoenix producing or HEK293T cells were used, respectively. Cells were plated on poly-D-lysine coated plates to achieve 80–90% confluency the following day. DNA was isolated with a DNA extraction kit (Qiagen) and eluted with sterile water. Producing cells were transfected with Turbofect (Thermofisher) according to manufacturer's protocols in serum free DMEM/F12 media. For lentiviral production packaging DNA (Addgene) was co-transfected with vector harboring transgene of interest. After cell growth overnight in DMEM/F12 media supplemented with 10% FBS, cells were washed with PBS and serum free DMEM/F12 was added for 24–36 hours. Harvested viruses were filtered using syringe driven 0.45 μM PVDF filters. Target cells were transduced with virus diluted 1:1 with fresh media in the presence of 8mg/ml of Polybrene (Sigma). At least two sequential infection were performed followed by cell selection with appropriate antibiotics. After 10–12 days of selection cells were harvested and transgene expression was verified by Western blot.

### NPC transformation and TW deletion

We previously transformed 3 month-old NPCs from C57Bl/6 females mouse forebrain using co-expression of HPVE6/7 and Ha-rasV12 [5]. To knockdown TW in these cells we stably infected a TW specific shRNA delivered by lentiviral vector [40] or corresponding empty vector into control cells. TWflox:mTmG NPCs were transformed by sequential retroviral transduction with HA tagged Ha-RasV12 cloned in LXSH vector followed by either myristoylated Akt1 cloned in neomycin resistant pWZL retroviral vector or shP53 mouse specific shRNA in puromycin lentiviral vector (Sigma) followed by selection with appropriate antibiotic. Akt/Ras transformed NPC cultures were split and transduced with a retroviral Cre recombinase construct or empty vector control (pBabe-Cre or empty pBabe vector) followed by selection with Zeocin. Animal implantations were performed from original unsorted cell pools or after cell sorting to eliminate contaminating unrecombined TW+ cells. Given the potential non-specific effects of constitutive Cre expression on tumor growth [33] we also used direct administration of Cre recombinase protein per the manufacturers protocol (Exellgen) to delete TW in wild-type cells (m Tomato+) followed by cell sorting to segregate of TW+ (red) and TW– (green) cells. Cre reporter activity was monitored by imaging detection of green or red epi-fluorescence.

### FACS analysis

To enrich for specific TW wild-type or TW null genotypes cells were isolated on the basis of mTmG Cre reporter expression using Aria 3 at Cell Sorting Core of the University of Washington. To eliminate confounding effects from residual TW expressing cells we performed serial FACS (F1 and F2) to effectively eliminate TW+ cells after the F2 isolation.

### Western blot analysis and protein stability assay

Western blots for TW and p53 were described [4, 5]. Equal amounts of proteins were separated on the SDS precast minigel (Biorad), transferred to the PVDF membrane and hybridized with primary antibody (p53 (Cell Signaling), TW (Santa Cruz Biotechnology), β-Actin (Sigma). Secondary HRP conjugated anti-rabbit or anti-mouse antibody and ECL reagent (Pierce) were used for antigen visualization.

P53 stability assay was performed as described previously [41]. Cells were treated with cycloheximide (CHX) (20 mg/ml) for 15, 30, 60, and 90 min, harvested lysed in cold RIPA buffer supplemented with protease inhibitors on ice and subjected to Western blotting. The p53 protein was quantitated using NIH ImageJ software and plotted as a percentage of p53 remaining. The average of two experiments was used to calculate the half-life of the p53 by regression analysis.

RT-PCR and qRT-PCR were performed as previously described [1, 2]. RNA isolation was performed using RNA easy kit (Qiagen) followed by RT reaction (Clontech). PCR was performed with SYBR Green mixture in 7900HT sequence detection system (Applied Biosystems) and analyzed using RQ Manager software. Primer sequences are provided upon request.

### Invasion assays

Invasion assays were performed using matrigel-coated filters (BD Biosciences) as previously described [2] except that the assay was performed in serum free media supplemented with 10 ng/ul and 40 ng/ul of EGF/bFGF in a top and bottom chamber, respectively.

### *In vivo* model and survival analysis

For the HPV/Ras studies where transformed NPCs were generated from C57Bl/6 mice we used 3mo syngeneic female C57Bl/6 mice as hosts. To mitigate logistic issues encountered in generating sufficient syngeneic hosts of the same age from the (129×1/SvJ × C57Bl/6) crosses we used immunocompromised nude mice (Taconic) for *in vivo* studies. A Hamilton syringe and computer driven injector attached to the Stereotactic frame (Stoelting) were used for intracranial implantation of equal numbers (150 thousand cells/mice) of transformed NPCs into the indicated host strains to assess tumor formation and effect on survival (at least 5 animals/group). Injection coordinates relative to Bregma were 2 mm lateral, 1.5 mm anterior and 3 mm ventral. Procedures for anesthesia, analgesia, injection, post-op recovery and monitoring were performed according to approved IACUC protocol. Mice were sacrificed when moribund demonstrating following clinical manifestations: rough hair coat (for syngeneic animals), hunched posture, decreased spontaneous activity, rapid shallow breathing or slow deep breathing, loss of body weight. For tissue collection, mice were deeply anesthetized with Avertin then transcardially perfused with PBS followed by 4% PFA. To assess the *in vivo* selection of TW+ and TW– cells with constitutive Cre expression, we injected nude hosts with 50:50 mixtures of TW+ and TW– cells as above and sacrificed animals at 10 and 20 days post injection without clinical manifestation.

### Slice culture experiments

Postnatal mouse brain slices were cultured according to established protocols [1] and TW+ expressing empty vector and TW– cells expressing constitutive Cre were seeded as 50:50 mixtures of 4 × 10^4^ cells. Cultures were analyzed by epi-fluorescent or confocal microscope at 2 and 6 days after seeding.

### Isolation of tumor derived cells

GFP expressing tumors were identified and dissected under a fluorescence dissecting microscope from unfixed tumor. GFP positive tumor fragments were digested with trypsin and plated in the media in the presence of a selection antibiotic to eliminate host-contaminating cells.

## SUPPLEMENTARY MATERIALS FIGURES



## Abbreviations

TW: Twist1
Rb: Retinoblastoma 1
Ras: Ha-Ras Transforming Protein P21
Akt: Protein Kinase B Alpha
HPV: Human papillomavirus
