# Supplementary material for: Twist1 mediated regulation of glioma tumorigenicity is dependent on mode of mouse neural progenitor transformation
Source: Oncotarget. 2017 Nov 21;8(64):107716–29. doi: 10.18632/oncotarget.22593 (PMC5746102; doi:10.18632/oncotarget.22593)
Supplement: Supplementary file 1 [file oncotarget-08-107716-s001.pdf]

Twist1 mediated regulation of glioma tumorigenicity is dependent on mode of mouse neural progenitor transformation

SUPPLEMENTARY MATERIALS

A

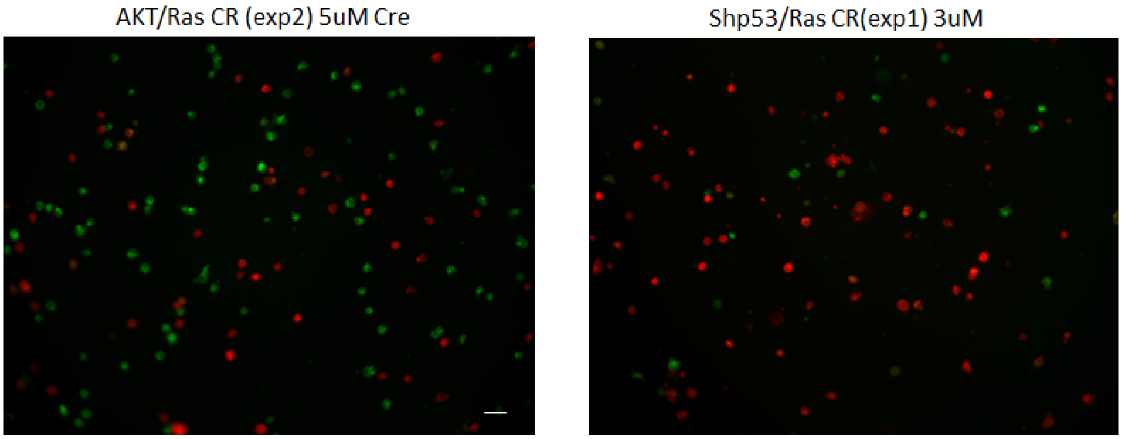

B

Cell line	GFP+	mTomato +	% GFP conversion
AR CR	873K	335K	72%
Rshp53 CR	44K	140K	24%

Supplementary Figure 1: Conversion of mTmG reporter in transformed NPCs by administration of recombinant Cre protein. (**A**) Representative images of Akt/Ras (left panel) and shP53/Ras (right panel) transformed NPCs after administration of indicated concentrations of recombinant Cre protein Scale bar, 50 µm. (**B**) Summary of conversion rates for the two transformed NPC cell cultures. Green cells indicate recombination events.

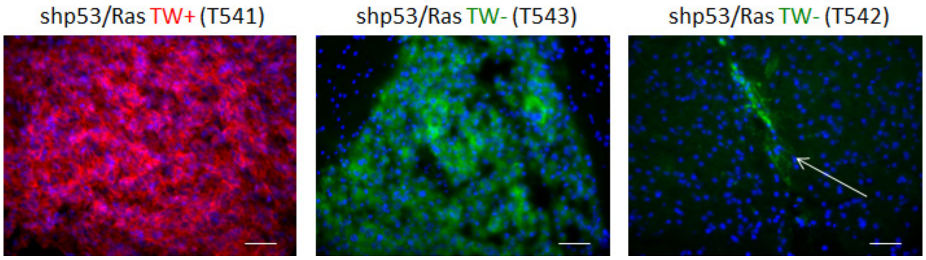

Supplementary Figure 2: shp53/Ras tumors retain TW genotype. Photomicrographs of representative shP53/Ras TW+ (red, left) and TW– (green, right) tumors showing stable retention of TW– cells in terminal tumors. Example of TW– micro-tumor from animal sacrificed at the end of experiment without clinical manifestation.
